# Substrate-dependent dynamics of the multidrug efflux transporter AcrB of *Escherichia coli*

**DOI:** 10.1038/srep21909

**Published:** 2016-02-26

**Authors:** Kentaro Yamamoto, Rei Tamai, Megumi Yamazaki, Takehiko Inaba, Yoshiyuki Sowa, Ikuro Kawagishi

**Affiliations:** 1 Department of Frontier Bioscience, Hosei University, Kajino-cho, Koganei, Tokyo 184-8584; 2 Research Center for Micro-Nano Technology, Hosei University, Midori-cho, Koganei, Tokyo 184-0003, Japan

## Abstract

The resistance-nodulation-cell division (RND)-type xenobiotic efflux system plays a major role in the multidrug resistance of gram-negative bacteria. The only constitutively expressed RND system of *Escherichia coli* consists of the inner membrane transporter AcrB, the membrane fusion protein AcrA, and the outer membrane channel TolC. The latter two components are shared with another RND-type transporter AcrD, whose expression is induced by environmental stimuli. Here, we demonstrate how RND-type ternary complexes, which span two membranes and the cell wall, form *in vivo*. Total internal reflection fluorescence (TIRF) microscopy revealed that most fluorescent foci formed by AcrB fused to green fluorescent protein (GFP) were stationary in the presence of TolC but showed lateral displacements when *tolC* was deleted. The fraction of stationary AcrB-GFP foci decreased with increasing levels of AcrD. We propose that the AcrB-containing complex becomes unstable upon the induction of AcrD, which presumably replaces AcrB, a process we call “transporter exchange.” This instability is suppressed by AcrB-specific substrates, suggesting that the ternary complex is stabilised when it is in action. These results suggest that the assembly of the RND-type efflux system is dynamically regulated in response to external stimuli, shedding new light on the adaptive antibiotic resistance of bacteria.

The increasing use of antibiotics has led to the emergence of multidrug resistant bacteria, a phenomenon that has serious consequences for public health. These problems are often caused by mobile genetic elements such as R plasmids, which contain various antibiotic resistance genes^1^. Some of these genes encode efflux transporters, which are classified into five superfamilies^2^,^3^: the major facilitator superfamily (MFS), small multidrug resistance (SMR), multidrug and toxic compound extrusion (MATE), ATP-binding cassette (ABC), and resistance-nodulation-cell division (RND). A frequent cause of multidrug resistance of gram-negative bacteria is elevated expression of multidrug efflux transporters of the RND type^4^. These systems comprise an inner membrane transporter (IMT), an outer membrane channel (OMC), and a membrane-fusion protein (MFP) that is anchored to the inner membrane via a lipid moiety and connects the IMT to the OMC^5^,^6^. This transporter superfamily is ubiquitous in nature and is found in prokaryotes and eukaryotes, including higher plants and animals^7^. The RND efflux systems of *Escherichia coli* and *Pseudomonas aeruginosa* have been extensively studied in terms of their biochemistry, molecular architecture, and patterns of gene expression^8^. Among the five RND transporters of *E. coli*, only the AcrB-AcrA-TolC complex (hereafter referred to as AcrBA-TolC) is constitutively expressed^9^ and plays a major role in its multidrug resistance^4^,^10^,^11^. The structure-function relationship of the IMT AcrB is well studied. The proton-drug antiporter AcrB^5^, which forms a homotrimer containing 12 transmembrane (TM) subunits^12^, captures a wide variety of antibacterial compounds, including antibiotics, detergents, and other amphiphilic agents, and directly transports these substrates out of the cell via TolC^10^,^13^. TolC is a homotrimeric protein consisting of an outer membrane domain folded into a 12-stranded β-barrel. It has a periplasmic extension (about 100 Å in length) with an α-helical coiled coil domain and a mixed α/β equatorial domain, which together form a hollow cylindrical structure that allows substrates to diffuse directly out of the cell^14^. Whereas the TolC homotrimer has three-fold symmetry^14^, the AcrB homotrimer is asymmetric, and each protomer plays a different role in substrate binding^15^. The functional association of AcrB with TolC is thought to require the MFP AcrA^16^, the stoichiometry of which has not been unambiguously determined. However, a recent cryo-EM study suggests that AcrA forms a homohexamer^17^.

The transcription of various inducible xenobiotic efflux transporter genes is regulated by two-component regulatory systems (TCSs)^18^,^19^, each typically consisting of a sensor kinase and a response regulator, which are widely distributed among prokaryotes and eukaryotes. The *E. coli* genome encodes 62 TCS proteins (*i.e.* sensor kinases and response regulators) that mediate a variety of environmental responses^20^,^21^. Most RND genes encoding MFP/IMT pairs, including *acrA/acrB*, are located in tandem on the chromosome and form transcriptional units (Supplementary Fig. S1a). Among all IMT genes, only the *acrD* gene stands alone. Its product, AcrD, is closely related to AcrB and also interacts with AcrA and TolC^22^, the latter of which is encoded by a gene belonging to a separate operon (Supplementary Fig. S1a). The AcrDA-TolC complex exports aminoglycosides and anionic β-lactams, such as carbenicillin and sulbenicillin, which are not transported by AcrB^23^,^24^,^25^. The expression of *acrD* is induced upon the addition of indole to culture media via the TCS consisting of the sensor kinase BaeS and the response regulator BaeR^26^,^27^.

These properties raise an important question concerning assembly of the ternary complex. Do newly synthesised AcrD molecules assemble into a ternary AcrDA-TolC complex *de novo*, or do they replace AcrB subunits in the preexisting AcrBA-TolC complex? It may be advantageous for bacteria to employ the latter mechanism, which we call “transporter exchange,” to remove harmful substrates out of cells as quickly as possible. However, it has not been established how RND-type transporter complexes that bridge two separate membranes are assembled. These considerations, and the pioneering study on single molecule behaviours of bacterial membrane proteins^28^, led us to study the assembly and dynamics of the AcrB/D AcrA TolC protein complex *in vivo*.

We visualised AcrB *in vivo* using green fluorescent protein (GFP). Observations with total internal reflection fluorescence (TIRF) microscopy revealed that most fluorescent foci of AcrB-GFP were stationary and mobile in the presence and absence of TolC, respectively. We next examined the effect of AcrD on the dynamics of AcrB. The fraction of mobile AcrB-GFP foci increased with increasing levels of AcrD. We therefore propose that the AcrBA-TolC complex becomes unstable upon the induction of AcrD, which presumably replaces AcrB in the ternary complex. Moreover, such instability is suppressed upon the addition of AcrB-specific substrates. These results suggest that the assembly of the RND-type efflux system is a regulated dynamic process that provides bacteria with a highly flexible repertoire of survival strategies to cope with a wide spectrum of antibiotics.

## Results

### The AcrB-GFP trimer is stationary in the cytoplasmic membrane via its association with TolC in the outer membrane

**Figure 1:**
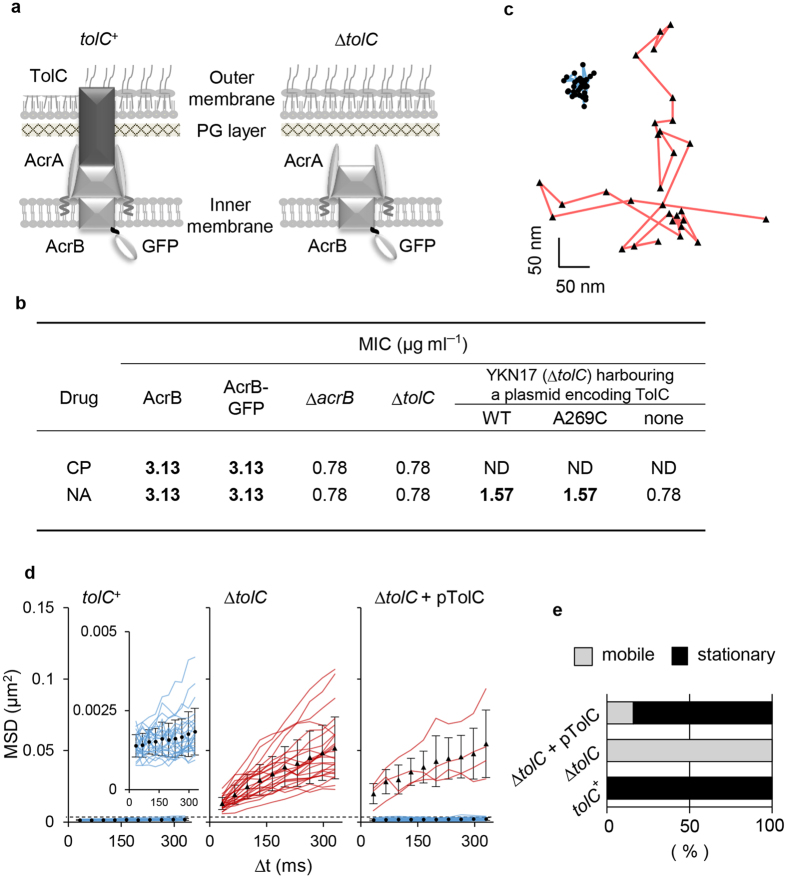
AcrB-GFP dynamics in the cytoplasmic membrane. (**a**) Schematic illustration of AcrB-GFP in the cell surface of *E. coli*. For simplicity, AcrB is depicted to bind AcrA even in the absence of TolC, though it has not been experimentally tested. (**b**) Antibiotic susceptibility analysis. Wild-type AcrB and AcrB-GFP were expressed from the chromosomal genes of strains BW25113 and YKN12, respectively, while plasmid-encoded wild-type TolC (pKRB2100) and mutant TolC (A269C; pKRB2104) were expressed in strain YKN17 (∆*tolC*). Bold face represents the resistance indicative of the significant efflux activity. Abbreviations: CP, chloramphenicol; NA, nalidixic acid; ND, not determined (due to the plasmid-borne CP resistance). (**c–e**) x-y trajectories of AcrB-GFP in YKN12 (blue line) and YKN17 (red line) with the TIRF illumination (**c**), MSD-∆*t* plots of AcrB-GFP foci (**d**) and fractions of mobile and stationary AcrB-GFP foci (**e**) in the presence or absence of TolC. Fluorescent foci were traced and their MSDs were calculated (*n* = 25). Closed symbols with error bars indicate averaged MSD values of all mobile (triangles) or stationary (circles) trajectories at each time with standard deviations. Dotted line in panel d indicates the boundary MSD value at time 330 ms to define mobile (red lines) and stationary foci (blue lines).

**Figure 2:**
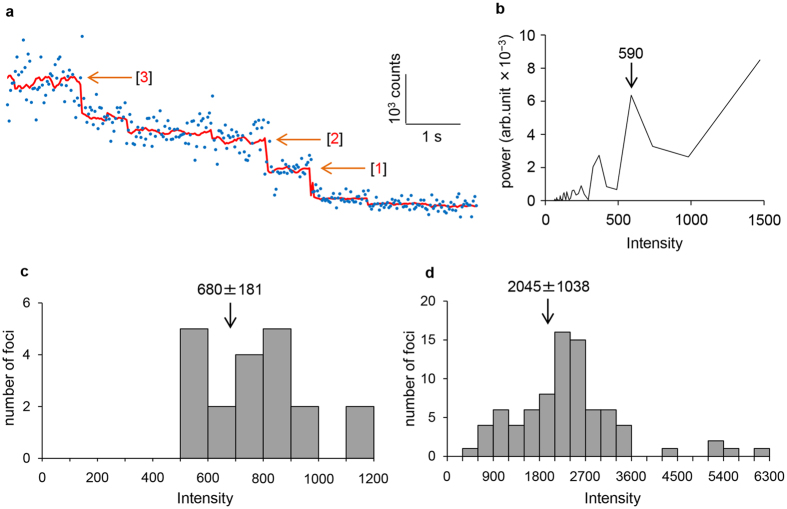
Trimeric nature of stationary AcrB-GFP foci in the presence of TolC. (**a**) Three-step photobleaching of a single AcrB-GFP focus. Blue dots, the intensity of AcrB-GFP per frame; red line, output of the edge-detecting filtered intensity (window = 15); orange arrows, the positions of predicted steps; and red numbers, bleaching step of AcrB-GFP (the values of step size are approximately 640, 640, and 560). (**b**) Power spectra of the PDDF with arrows indicating step sizes of photobleaching trace. (**c**) Distribution of the detected step sizes of photobleaching (*n* = 20). Arrow indicates an average (mean ± S.D.) of the step sizes. (**d**) Distribution of the fluorescence intensity of single foci at the first frame obtained by subtracting the background value (*n* = 81). Arrow indicates an average (mean ± S.D.) of the foci intensity.

We constructed a strain expressing AcrB fused to green fluorescent protein from the chromosomal *acrB* locus (strain YKN12, a derivative of strain BW25113^29^). The AcrB-GFP protein retained almost full activity as judged by the minimal inhibitory concentrations (MIC) of AcrB substrates (Fig. 1b). To assess the effect of TolC on the AcrB-GFP dynamics within the cell membrane, we constructed a *tolC*-deleted derivative of YKN12 (strain YKN17). Immunoblotting with monoclonal anti-GFP antibody detected a band of AcrB-GFP without visible degradation products (Supplementary Fig. S2). The expression level of the AcrB-GFP in a *tolC*-deleted strain was almost the same as that of the *tolC*^+^ strain (Supplementary Fig. S2). TIRF microscopic observations detected a clear difference in the lateral displacements of AcrB-GFP foci between strains YKN12 (*tolC*^+^) and YKN17 (∆*tolC*) (Fig. 1a,c). Figure 1c shows the x-y trajectories of AcrB-GFP foci in these genetic backgrounds. AcrB-GFP movement (an AcrB subunit with 12 TMs has a molecular mass of 110 kDa) was analysed by monitoring two-dimensional mean square displacements (MSD) of individual foci over time. In the presence of TolC, the calculated MSD values of all AcrB-GFP foci tested at time 330 ms were distributed below 0.5 × 10^−2^ μm^2^, with an average of 0.2 ± 0.1 (mean ± S.D.) × 10^−2^ μm^2^ (Fig. 1d, Supplementary Fig. S3a and Supplementary Video. S1). In the absence of TolC, most AcrB-GFP foci moved incessantly, and their MSD values were distributed over a wide range: 2.3−10.7 × 10^−2^ μm^2^, with an average of 5.2 ± 2.2 (mean ± S.D.) × 10^−2^ μm^2^ (Fig. 1d, Supplementary Fig. S3a and Supplementary Video. S2). We then fitted the data (*i.e.,* the averaged MSD-∆*t* plots for stationary and mobile fractions of AcrB-GFP in strains YKN12 and YKN17) to linear regression models (Supplementary Fig. S3b). The diffusion coefficient (= *D*) values in the presence and the absence of TolC calculated from these fits are 6.8 ± 5.0 (mean ± S.D.) × 10^−4^ μm^2^ s^−1^ and 3.5 ± 1.8 × 10^−2^ μm^2^ s^−1^, respectively. When complemented with a plasmid encoding TolC, MSD values of AcrB-GFP foci and resistance to nalidixic acid returned to levels comparable to those of the parent strain (Fig. 1b,d and Supplementary Video. S3). AcrB-GFP foci in the *tolC*^+^ and the complemented strains showed about two orders of magnitude smaller *D* values (8.0 ± 4.0 × 10^−4^ μm^2^ s^−1^) than those in the ∆*tolC* strain. The former can be regarded as immobile within experimental error. Based on these data, foci with MSDs at time 330 ms below and above 0.5 × 10^−2^ μm^2^ (marked with a dotted line in Fig. 1d) will hereafter be designated as “stationary” and “mobile,” respectively. Accordingly, all the AcrB-GFP foci in the *tolC*^+^ background are stationary and those in the ∆*tolC* background are mobile, where as 84% of those in the complemented strain are stationary (Fig. 1e). In the *tolC*^+^ background, time-course analyses of the fluorescence emission from single stationary AcrB-GFP foci detected up to three-step photobleaching (Fig. 2a). Figure 2b shows the power spectrum of one focus as the pairwise differences (Pairwise Difference Distribution Function, PDDF), which indicate the step size of a single GFP molecule, with an average of 680 ± 180 (mean ± S.D., arbitrary units) under our experimental conditions (Fig. 2b,c and Supplementary Fig. S4). The distribution of intensity at the first frame peaked at a value that is about three times the intensity of a single GFP estimated by photobleaching (Fig. 2d).

### Visualisation of TolC with a fluorescent reagent

**Figure 3:**
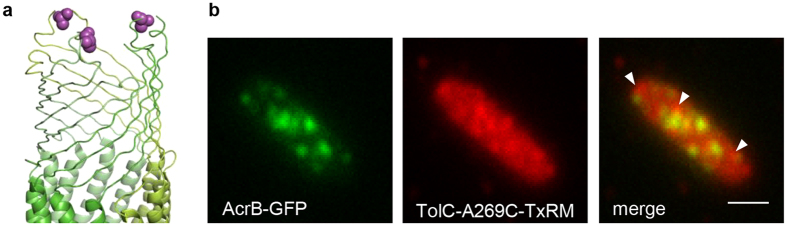
The co-localisation of AcrB-GFP foci with fluorescently labelled TolC. (**a**) The crystal structure of the extracellular loop of the TolC trimer (PDB ID code: 2XMN). Purple balls indicate the positions of A269C. (**b**) Co-localisation of AcrB and TolC. Images of AcrB-GFP (left) and TxRM-labelled TolC-A269C (middle) were merged (right). Arrow heads indicate TolC-A269C foci that did not co-localise with AcrB-GFP. Scale bar, 1 μm.

We next examined whether stationary AcrB-GFP foci in the inner membrane indeed co-localise with TolC in the outer membrane. Substituting Cys for Ala-269 in an extracellular loop of TolC (11 residues in length; Fig. 3a) did not affect drug efflux activity as judged from MIC of nalidixic acid (Fig. 1b). TolC-A269C was stained with the thiol-reactive fluorescent reagent Texas Red maleimide (TxRM) at low concentrations for a short period of time to minimise non-specific labelling of other Cys-containing proteins. TIRF microscopy detected foci of labelled TolC-A269C, some of which co-localised with AcrB-GFP (Fig. 3b). No foci were observed in cells expressing wild-type TolC, demonstrating that non-specific labelling was negligible (Supplementary Fig. S5).

### AcrB dissociates from the preformed complex with TolC and AcrA upon induction of AcrD

**Figure 4:**
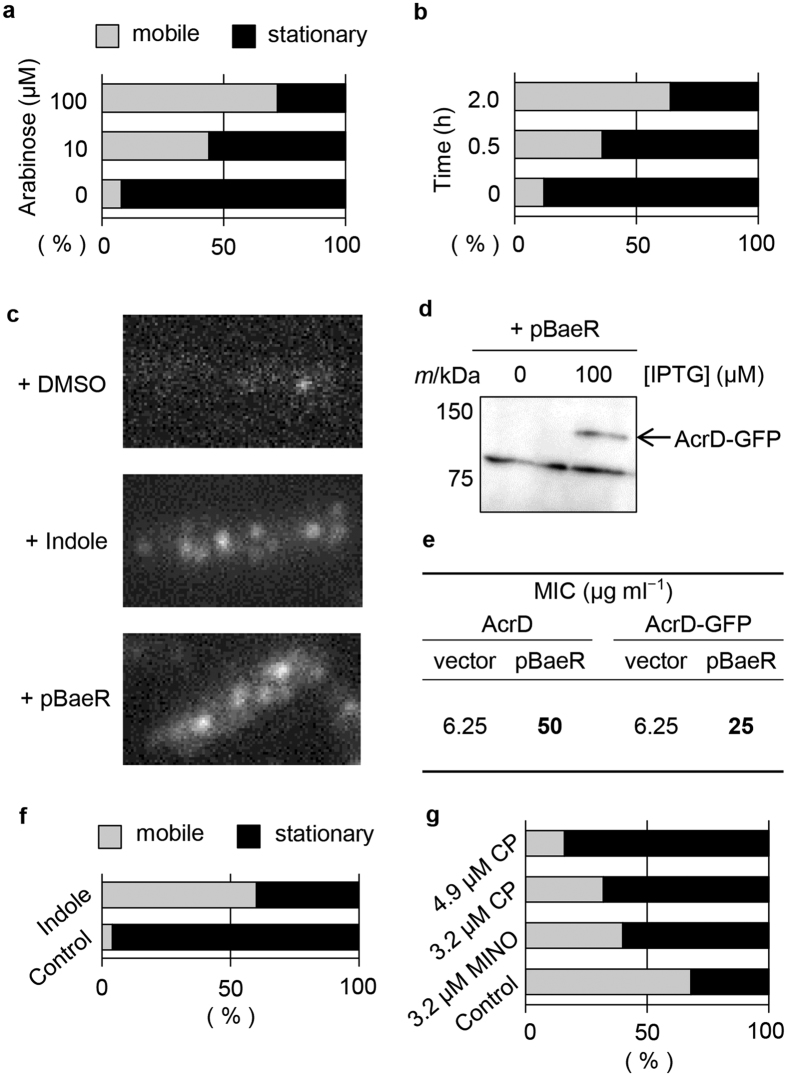
The AcrD expression influenced the mobility of AcrB-GFP foci. (**a,b**) Effect of plasmid-encoded AcrD induced with 0−100 μM arabinose for 2 h (*n* = 25) (**a**) or with 100 μM arabinose for 0−2 h (*n* = 25) (**b**). (**c,d**) TIRF observation (**c**) and immunodetection (**d**) of AcrD-GFP (*m*/kDa = 140) in MBRT02 cells. (**e**) Resistance of cells expressing AcrD (strain BW25113) or AcrD-GFP (strain MBRT02) to carbenicillin. Cells carrying the BaeR-expressing plasmid pBaeR or the vector pCA24N were cultured with 100 μM IPTG. (**f**) Fractions of stationary and mobile AcrB-GFP foci with or without 4 mM indole (*n* = 25). (**g**) AcrB-specific substrates stabilise the association of AcrB-GFP foci with TolC. Fractions of stationary and mobile AcrB-GFP foci under the expression of AcrD in the presence or absence of chloramphenicol (CP, 4.9 or 3.2 μM) and minocycline (MINO, 3.2 μM). Plasmid-encoded AcrD was induced with 100 μM arabinose for 2 h (*n* = 25).

We were interested in determining whether the expression of AcrD, the closest homolog of AcrB, influences AcrB dynamics in the *tolC*^+^ strain. First, AcrD was expressed from an arabinose-inducible plasmid. The fraction of mobile AcrB-GFP foci increased (from 8% to 72%) with increasing concentrations (0−100 μM) of arabinose (Fig. 4a, Supplementary Fig. S6a) and increased (from 12% to 64%) over time (0−2 h) after the addition of 100 μM arabinose (Fig. 4b, Supplementary Fig. S6b). We suggest that the induction of AcrD facilitates dissociation of AcrB from the preformed complex with TolC and AcrA, presumably to form a new ternary complex (AcrDA-TolC). We then tested whether the dynamics of AcrB-GFP is influenced by the expression of AcrD from the native chromosomal gene. The expression of *acrD* was induced by the addition of indole or by overexpressing the TCS response regulator BaeR^26^,^27^. We next constructed a strain (named MBRT02) carrying a chromosomal gene encoding AcrD-GFP (the design is essentially the same as *acrB*-*gfp* in strain YKN12). In strain MBRT02, AcrD-GFP foci were observed when cells were exposed to indole or when BaeR was overexpressed from the plasmid (Fig. 4c). The increased expression of AcrD-GFP induced by plasmid pBaeR was verified by immunoblotting with monoclonal anti-GFP antibody (Fig. 4d). Consistent with that result, the fraction of mobile AcrB-GFP foci in YKN12 cells treated with indole (60%) was larger than that in cells not exposed to indole (4%) (Fig. 4f and Supplementary Fig. S6c). Taken together, these results demonstrate that transporter exchange occurs in the native setting.

### The AcrBA-TolC complex is stabilised by AcrB-specific substrates

The findings just described raise the question of whether the induction of AcrD destabilises the AcrBA-TolC complex while the latter complex is in the process of exporting its substrates. AcrBA-TolC, but not AcrDA-TolC, exports chloramphenicol and minocycline. When either of these substrates was added to the culture medium, the fraction of stationary AcrB-GFP foci became relatively insensitive to the expression of AcrD (32% without a substrate *vs* 84% with 4.9 μM chloramphenicol and 60% with 3.2 μM minocycline; Fig. 4g and Supplementary Fig. S6d). This was further examined under more physiological conditions: indole-induced expression of AcrD-GFP in MBRT02 cells. The functionality of the AcrD-GFP protein in these experiments was verified by checking the MIC for an AcrD-specific substrate, carbenicillin (Fig. 4e). The AcrB-specific substrate chloramphenicol decreased the fraction of stationary AcrD-GFP foci (Supplementary Video. S4). We also examined whether the incorporation of IMT molecules into the ternary complex is affected by its substrate. The addition of the AcrD-specific substrate carbenicillin to cells expressing AcrD from the plasmid did not reduce the fraction of stationary AcrB-GFP (32% without a substrate *vs* 36% with carbenicillin; Supplementary Fig. S7). These results suggest that the AcrD-induced instability of the preformed AcrB-containing complex is suppressed when the preexisting complexes are transporting substrates, whereas the presence of a substrate of a second, newly synthesised transporter does not necessarily facilitate transporter exchange.

## Discussion

In this study, we examined the dynamics and assembly of AcrB, the major RND-type xenobiotic efflux transporter in the inner membrane of *E. coli*. With TIRF microscopy, we found that most foci of GFP-fused AcrB were stationary in the presence of TolC, whereas they showed lateral diffusion in the membrane of the Δ*tolC* strain. The co-localisation of AcrB-GFP with TolC was detected by chemically labelling TolC. We also found that the induction of AcrD destabilises the AcrBA-TolC complex, presumably resulting in the exchange of AcrB-GFP in the preexisting complex with newly synthesised AcrD. The fraction of mobile AcrB-GFP foci increased with increasing expression levels of AcrD. Furthermore, the AcrD-induced instability of the AcrBA-TolC complex was suppressed by AcrB-specific substrates, suggesting that the assembly of the RND-type efflux system is a regulated dynamic process.

The *D* value of mobile AcrB-GFP obtained in our TIRF microscopic analyses (3.5 ± 1.8 × 10^−2^ μm^2^ s^−1^) is roughly one order of magnitude smaller than that of the *E. coli* serine chemoreceptor Tsr fused to the fluorescent protein Venus (4.0 ± 0.1 × 10^−1^ μm^2^ s^−1^)^30^. The difference in the *D* value between these proteins is reasonable considering that in the number of TMs: AcrB is a homotrimer of 12-TM subunits and Tsr is a homodimer of 2-TM subunits (or exists as a trimer of dimers). The fact that AcrB-GFP foci are stationary in the presence of TolC is consistent with the structural features of TolC. Because the periplasmic extension of TolC penetrates the peptidoglycan layer, a rigid three-dimensional mesh-like supramolecule, the lateral diffusion of TolC molecules must be very restricted. Thus, the diffusion of AcrB, once assembled into a complex with TolC, must also be limited.

We detected three-step photobleaching of stationary AcrB-GFP foci. We also detected double-sized steps, indicating that two GFP molecules were bleached simultaneously or successively. These results demonstrate that at least a majority of TolC-associated AcrB molecules form trimers. The broad distribution of MSD value in the absence of TolC might reflect interactions with other membrane proteins or a mixed population of AcrB-GFP monomers and oligomers.

Leake *et al*. (2006) reported that TIRF illumination visualises approximately one sixth of the surface of a cell within 100 nm of a coverslip^28^. In the *tolC*^+^ strain, we detected an average of 7.5 ± 1.6 (mean ± S.D., *n* = 50) stationary foci of AcrB-GFP per cell in the TIRF illumination field. We therefore estimate that there are 45 ± 9.6 foci of AcrB-GFP trimers per cell, which corresponds to 135 ± 28.8 molecules of AcrB-GFP per cell if a majority of foci represented trimers. This estimate is consistent with estimated values the literature (<500 molecules)^31^. Because the number of TolC molecules per cell is nearly three times higher than that of AcrB molecules^31^, and because the level of TolC expressed from the plasmid-borne gene must be higher than from the chromosomal gene, it is likely that not all labelled TolC co-localised with AcrB-GFP and that TolC is in excess.

Expression of the *acrAB* operon is regulated by AcrR repressor and transcriptional activators such as MarR and SoxS^32^,^33^,^34^. However, the operon is known to be “constitutively” expressed under laboratory conditions^9^, whereas genes encoding other IMTs, including AcrD, of RND-type xenobiotic efflux systems are expressed at very low levels in *E. coli* and are induced by environmental stimuli such as indole-induced and other stresses on the cell envelope^27^,^35^. Because AcrD shares TolC and AcrA with AcrB, and assuming that the induction signal for AcrD (indole) does not significantly affect the cellular amounts of AcrB, AcrA and TolC, it was reasonable to ask whether AcrD can replace AcrB in the preformed AcrBA-TolC complex. Indeed, the expression of AcrD either from the chromosomal gene with the native promoter (induced by indole) or the promoter-less gene under the control of the *araBAD* promoter (induced by arabinose) increased lateral diffusion of AcrB-GFP, which was otherwise stationary in a complex with TolC and AcrA. This AcrD-induced instability of the AcrBA-TolC complex, which may result in transporter exchange, would be part of adaptive drug resistance, an important bacterial response to harmful substances. It should also be noted that transporter exchange may occur between different molecules of the same IMT: for instance, the AcrBA-TolC complex may undergo assembly and disassembly with some frequency. Thus, an RND-type xenobiotic efflux complex is not rigid or fixed but allows association and dissociation of its components.

The next obvious question is whether the AcrD-induced instability of the AcrBA-TolC complex can be regulated. Our results indicate that there is some level of regulation: the AcrBA-TolC complex was stabilised by the AcrB-specific substrates. Of the two substrates tested, minocycline showed a weaker effect, likely because it has a lower affinity for AcrB. Minocycline might also be sequestered because it chelates divalent metal ions in the medium^36^. These results suggest that the AcrBA-TolC complex is stabilised by substrate binding, slowing the exchange of AcrB with AcrD. The stabilisation of a ternary complex when it is in action should be advantageous for bacterial survival. In particular, AcrB can transport the widest range of substrates among all xenobiotic transporters^6^, including the bacterium’s own metabolites or their derivatives such as fatty acids and bile salts^37^. Interference with the activity of AcrB when cells are exposed to its substrate(s) would presumably have adverse effects on viability^38^. The AcrD-specific substrate, however, had little effect on the stability of the ternary complex. Thus, the presence of a substrate for a non-associated IMT may not necessarily accelerate destabilisation of preexisting ternary complexes. Alternatively, a free IMT might not bind its substrate. A thermodynamics study suggested that, in the absence of substrate, AcrB forms a symmetric trimer, and that forced substrate binding to one protomer induces and stabilises an asymmetric form of the AcrB trimer^39^. *In vivo*, an IMT might bind its substrate to become an asymmetric trimer only when it is in the ternary complex. The resulting asymmetric IMT trimer might stabilise the IMT-MFP-OMC ternary complex, making it resistant to transporter exchange. The AcrBA-TolC complex might also be stabilised by some endogenous substrate(s).

**Figure 5:**
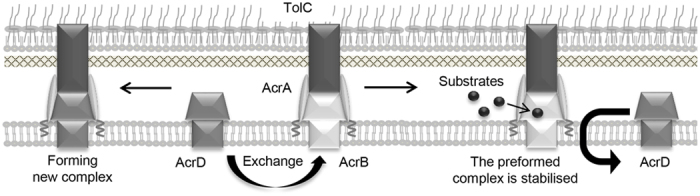
The transporter exchange model. The preformed AcrB-AcrA-TolC complex becomes instable upon the induction of AcrD, which presumably result in the exchanges of AcrB with AcrD to form a new AcrDA-TolC complex (left). The preformed ternary complex can be stabilised by its substrates (right).

In conclusion, we propose that newly synthesised transporter molecules can destabilise preexisting RND-type efflux complexes to facilitate transporter exchange. This exchange is suppressed by substrates of the transporter that is already in the complex (Fig. 5). This mechanism may enable a rapid adaptive resistance to a newly encountered toxic compound. Increasing the instability of the RND-type efflux complex could facilitate the development of effective cocktails of antibiotics that are substrates for different drug efflux systems to overcome drug resistance by gram-negative bacteria.

## Materials and Methods

### Strains and plasmids

Bacterial strains and plasmids used in this study are described in Supplementary Table S1.

### Plasmids

The plasmid vector pTrcHisB (Invitrogen) carries the *trc* promoter, *lacI*^q^ and *bla*. The pTrcHisB-derived plasmid pDS1050 encodes Gly_3_−linked−GFP^40^. The plasmids vectors pBAD24 and pBAD33 carry *bla* and *cat*, respectively, in addition to the *araBAD* promoter and *araC*, which encodes the regulator of the *araBAD* promoter^41^.

### Construction of plasmids encoding wild-type or GFP-fused IMT

The *acrB*- or *acrD*-coding region was amplified using PCR to introduce *Nhe*I and *Bgl*II sites at their 5ʹ and 3ʹ ends, respectively. The resulting fragment was cloned between the *Nhe*I and *Bgl*II sites of the vector pDS1050 to yield plasmids encoding AcrB-GFP (pKRB2000) or AcrD-GFP (pKRB2010). For tight regulation of *acrD* expression, plasmid pBAD24 was used as a vector to clone a PCR-amplified coding region with *Nco*I and *Hin*dIII sites at its 5ʹ and 3ʹ ends, respectively, to yield pKRB2050. The 3.3-kb *Mlu*I-*Hin*dIII fragment of pKRB2050 was cloned between the corresponding sites of plasmid pBAD33 to yield pKRB2053.

### Construction of plasmids encoding wild-type or mutant TolC

A *tolC*-expressing plasmid was constructed by cloning a PCR-amplified fragment containing the 5ʹ-untranslated and the coding regions of *tolC* between the *Sac*I and *Hin*dIII sites of the vector pBAD33 to yield plasmid pKRB2100 encoding wild-type TolC. The upstream primer used was designed to cover 28 bases just upstream of the deduced Shine-Dalgarno sequence of chromosomal *tolC*. A derivative of pKRB2100 encoding TolC-A269C (pKRB2104) was constructed using the QuickChange II one-day site-directed mutagenesis method (Stratagene) with Pyrobest DNA Polymerase (Takara Bio) and *Dpn*I (New England Biolabs).

### Construction of strains YKN12 and MBRT02 with chromosome-encoded AcrB-GFP and AcrD-GFP, respectively

The stop codon of *acrB* or *acrD* was replaced with a sequence encoding Gly_3_-GFP followed by a stop codon (Supplementary Fig. S1b). Strains were constructed using a λ Red recombination system with plasmid pKD46 encoding the Red recombinase^29^ and positive selection for the loss of tetracycline resistance^42^. To replace the stop codon of *acrB* or *acrD* on the chromosome of the standard strain BW25113 with the selectable tetracycline-resistance gene (*tetRA*), we designed primers with 40-nucleotide arms and generated fragments using PCR with plasmid pKRB2000 or pKRB2010 as the template. After recombination, selection and isolation, *tetRA* was replaced by a Gly_3_-GFP-encoding fragment with 40-nucleotide arms at their 5ʹ and 3ʹ ends. Tetracycline-sensitive clones were selected using tetracycline-sensitive medium (1% Bacto Tryptone, 0.5% Yeast Extract, 1% NaCl, 0.2% glucose, 50 μg mL^−1^ chlortetracycline hydrochloride, 1% NaH_2_PO_4_-H_2_O, 12 μg mL^−1^ fusaric acid, and 0.1 mM zinc chloride).

### Construction of the *tolC*-deletion strain YKN17

The *tolC* deletion was introduced using P1 transduction with strains JW5503^43^ and YKN12 as a donor and a recipient, respectively. After the selection on medium containing kanamycin and sodium citrate, transductants were purified twice.

### Analyses of single molecule behaviours using TIRF microscopy

Cells were grown in TG medium [1% Bacto Tryptone, 0.5% NaCl and 0.5% (w/v) glycerol] supplemented with appropriate antibiotics with shaking at 30 °C for 16 h. The overnight cultures were diluted 100-fold into fresh TG medium and incubation was continued for 4 h. Cells were harvested and washed twice with MLM buffer (10 mM potassium phosphate buffer pH7.0, 0.1 mM potassium EDTA pH7.0, 10 mM sodium lactate pH7.0 and 0.01 mM l-methionine).

The expression of AcrD-GFP or AcrD form the chromosomal gene was induced by the addition of indole or introduction of a plasmid encoding BaeR (pCA24N-baeR, pBaeR)^44^. Cells were grown in TG medium supplemented with appropriate antibiotics with shaking at 30 °C for 16 h. The culture was diluted 100-fold into fresh TG medium and further cultured for 2 h, and then 1% DMSO (negative control), 4 mM indole or 100 μM isopropylthiogalactoside (IPTG) were added to the culture, which was then incubated for 2 h. Cells were harvested and washed twice with MLM buffer.

Experiments to test the stability of the ternary complex were conducted using plasmid pKRB2050 carrying the wild-type *acrD* coding region downstream of the arabinose-inducible *araBAD* promoter. YKN12 cells (*acrB-gfp*) transformed with pKRB2050 were grown for 16 h with shaking at 30 °C in TG medium supplemented with ampicillin. Cultures were diluted 100-fold into fresh TG medium and cultured for another 2 h. Arabinose at appropriate concentrations was then added to the culture and incubation continued for another 2 h. The *tolC* complementation test was performed with induction of the plasmid-borne *tolC* at 100 μM arabinose. Cells (∆*tolC*) transformed by plasmid pKRB2100 were harvested and washed twice with MLM buffer. When necessary, antibiotics were added at appropriate concentrations 1.5 h after the addition of arabinose.

Cells were spotted onto a poly-l-lysine-coated coverslip, washed with MLM buffer and were observed using objective-type TIRF microscopy^45^,^46^ with an Olympus IX71 equipped with a 100× oil-immersion objective lens, lasers and a dichroic mirror (Semrock Di01-R488-561). GFP was visualised using a Cobolt Blues 50 mW laser (Cobolt 0473-04-01-0050-300) and emission filter (Semrock FF01-520/35-25). TxRM was visualised using a Cobolt Jive 50 mW laser (Cobolt 0561-04-01-0050-300) and emission filter (Semrock FF01-593/40-25). Images were recorded with 33 ms exposure using an EMCCD camera (Andor iXon DU-897). All of the image analysis was performed with the ImageJ ver. 1.48 software (NIH) and custom LabVIEW programmes. For tracking single foci, each images were filtered with the rolling ball algorithm (*Rolling Ball Radius*: 12 pixels) to subtract the background intensity. We defined a 7 × 7 pixel (350 × 350 nm) region of interest (ROI) centred on a focus in the first frame of the images with the threshold appropriately adjusted^28^. Individual foci were tracked through >30 frames. The mean square displacements (MSDs) were calculated as described in the literature^47^. The diffusion coefficients (=*D*) were calculated from the values of averaged MSD-∆*t* plots with linear regressions fitting of the first ten points.

### Estimation of the number of AcrB-GFP molecules per focus using three-step photobleaching

The analysis was carried out essentially as described in the literature^28^. In images recorded with the exposure times of 33 ms for 10 s using TIRF microscopy, fluorescent intensity per frame of a ROI centred at a fluorescent focus was monitored over time. The edge-detecting method of non-linear filtering (window = 15) was used to identify photobleaching steps in the time-course of AcrB-GFP intensity^28^,^48^.

### Fluorescent labelling of TolC-A269C

YKN17 cells (∆*tolC*) transformed with plasmid pKRB2100 encoding TolC-A269C or the vector pBAD33 (a negative control) were grown in TG medium at 30 °C for 16 h. The culture was diluted 100-fold into fresh TG medium and cultured for 2 h, and then 10 μM arabinose were added to the culture, which was further incubated for 2 h. Washed cells were treated with 1 μM TxRM (Texas Red C_2_ Maleimide, Molecular Probes) at room temperature for 5 s and then quickly washed twice with MLM before observation by TIRF microscopy. Images were averaged (10 frames) using the ImageJ software with custom macros.

### Antibiotic susceptibility analysis

Minimum inhibitory concentrations (MICs) were determined on YT agar plates^6^ (0.8% Bacto Tryptone, 0.5% Yeast Extract, 0.5% NaCl, 1.5% Bacto Agar) containing antibiotics at various concentrations. Bacteria were grown in YT medium at 37 °C for 16 h. The culture was diluted 100-fold into fresh YT medium supplemented with 100 μM arabinose or 100 μM IPTG, if necessary, and cultured further. YT agar plates with antibiotics and appropriate inducers, if necessary, were inoculated with aliquotss of cell suspension (2.5 μL each containing 10^5^ cells) and then incubated at 37 °C for 18 h.

### Immunoblotting

The overnight culture was diluted 100-fold into fresh TG medium and further cultured for 4 h. Immunoblotting using a monoclonal anti-GFP antibody (Nacalai Tesque). Immune complexes were detected using horseradish-peroxidase (HRP)-labelled anti-mouse IgG (KPL) and the Immobilon Western Chemiluminescent HRP Substrate system (Merck Millipore).

## Additional Information

**How to cite this article**: Yamamoto, K. *et al.* Substrate-dependent dynamics of the multidrug efflux transporter AcrB of *Escherichia coli. Sci. Rep.* **6**, 21909; doi: 10.1038/srep21909 (2016).

## Supplementary Material

Supplementary Information

Supplementary Video S1

Supplementary Video S2

Supplementary Video S3

Supplementary Video S4
