# Supplementary material for: Substrate-dependent dynamics of the multidrug efflux transporter AcrB of Escherichia coli
Source: Sci Rep. 2016 Feb 26;6:21909. doi: 10.1038/srep21909 (PMC4768149; doi:10.1038/srep21909)
Supplement: Supplementary Information [file srep21909-s1.pdf]

Supplementary Information

Substrate-dependent dynamics of the multidrug efflux transporter AcrB of *Escherichia coli*

Kentaro Yamamoto, Rei Tamai, Megumi Yamazaki, Takehiko Inaba, Yoshiyuki Sowa, and Ikuro Kawagishi

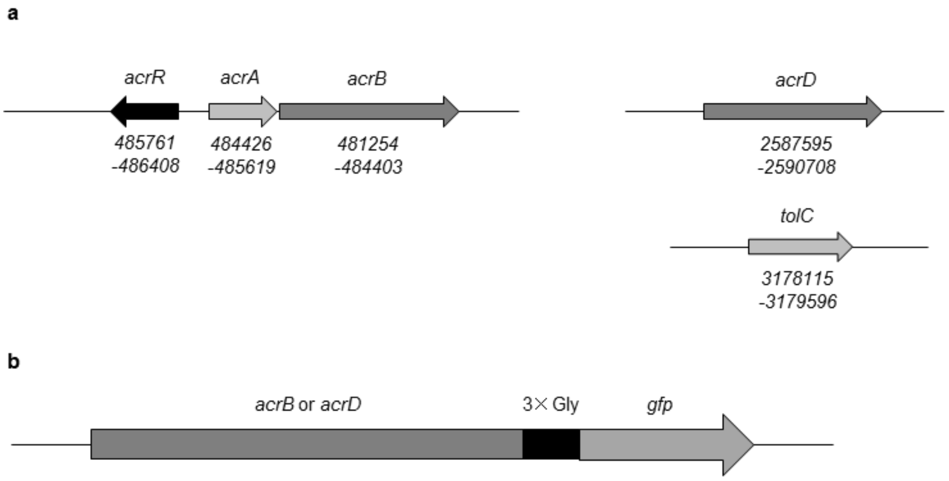

Figure S1 | Characterisation of AcrB-GFP, AcrD-GFP, and mutant TolC. (**a**) The loci of RND-component genes on the chromosome of *E. coli* str. K-12 substr. MG1655. (**b**) Construction of AcrB-GFP and AcrD-GFP. GFP was fused to the carboxy-terminus of AcrB or AcrD through a Gly_3 linker.

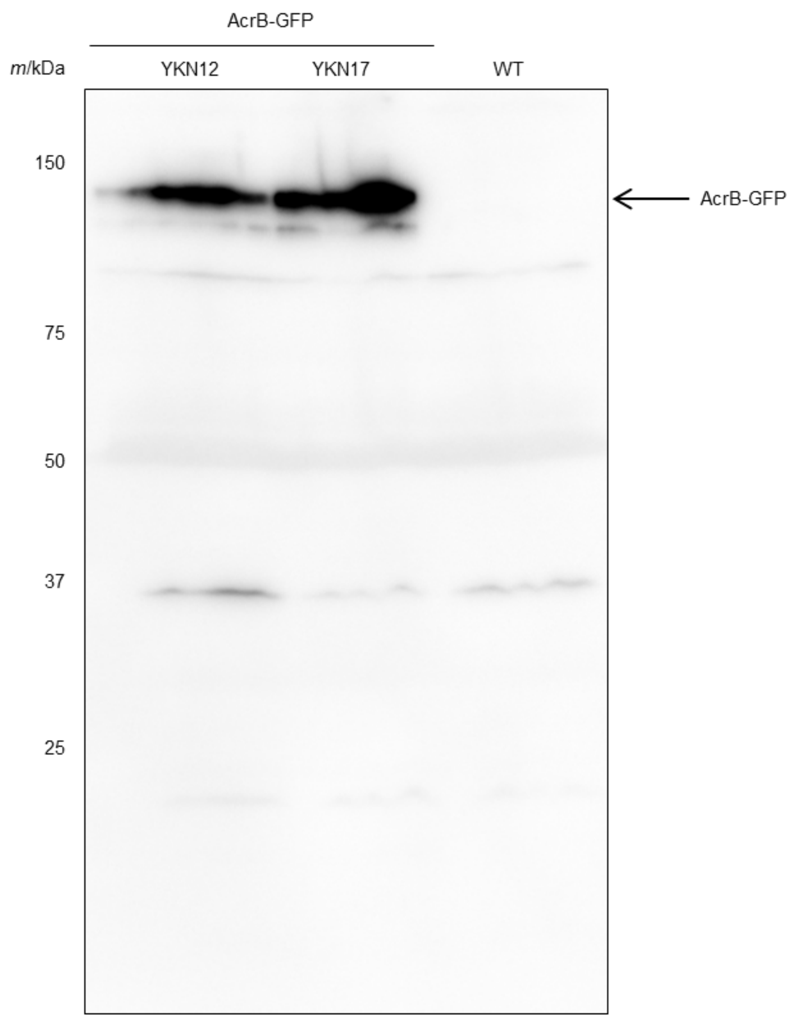

Figure S2 | Detection of AcrB-GFP using immunoblotting. AcrB-GFP (*m*/kDa = 140) was expressed in strains YKN12 (*tolC*$^{+}$) (left lane) and YKN17 (Δ*tolC*) (middle lane). The wild-type strain BW25113 was used as a control (right lane). Whole cell lysates were subjected to Western blotting with monoclonal antibody raised against GFP and anti-mouse-IgG (Cell Signaling Technology) antibody labelled with horse-radish peroxidase.

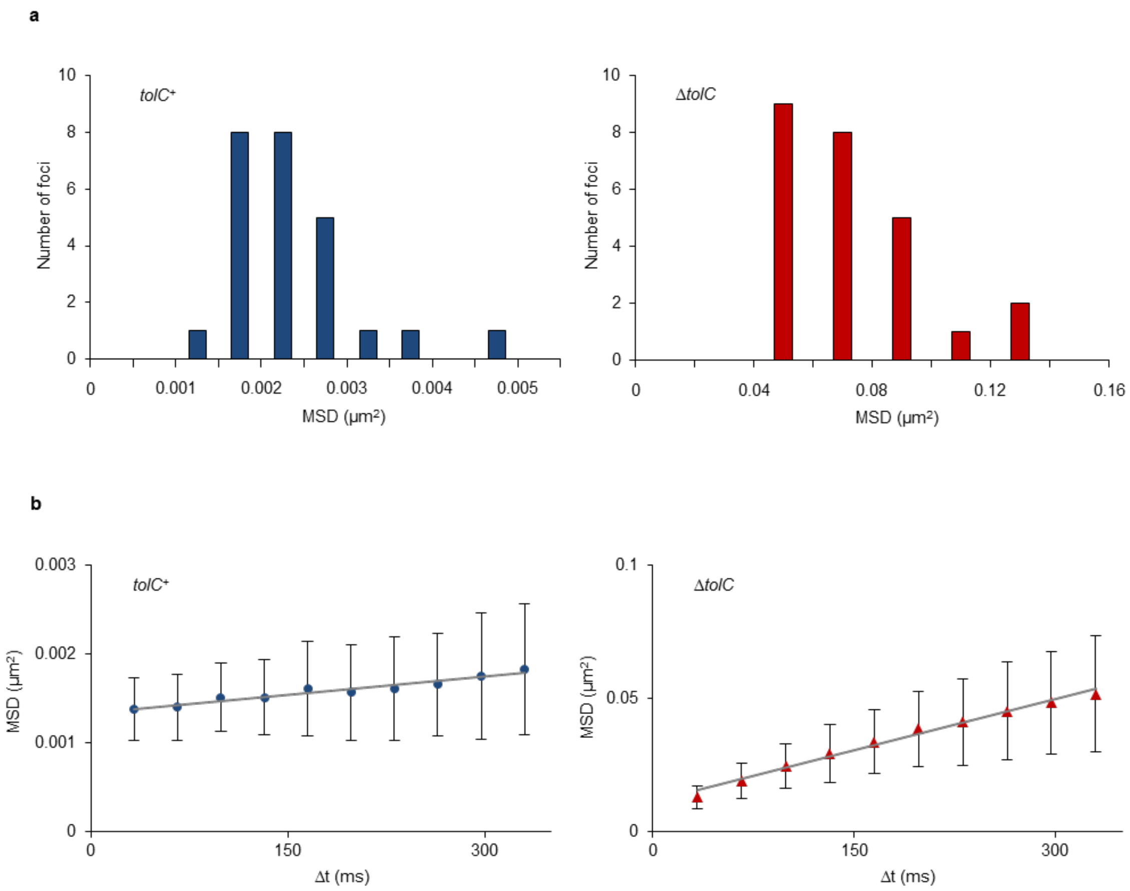

Figure S3 | Tracking AcrB-GFP foci and calculation of their mean square displacements (MSD) in strains YKN12 (*tolC*$^{+}$) and YKN17 (Δ*tolC*). (**a**) Distribution of MSD at time 330 ms of AcrB-GFP foci in the presence (left) or absence (right) of TolC. (n = 25). (**b**) The averaged MSD-Δt plots for stationary (left) and mobile (right) fractions of AcrB-GFP. These data were fitted with linear regressions.

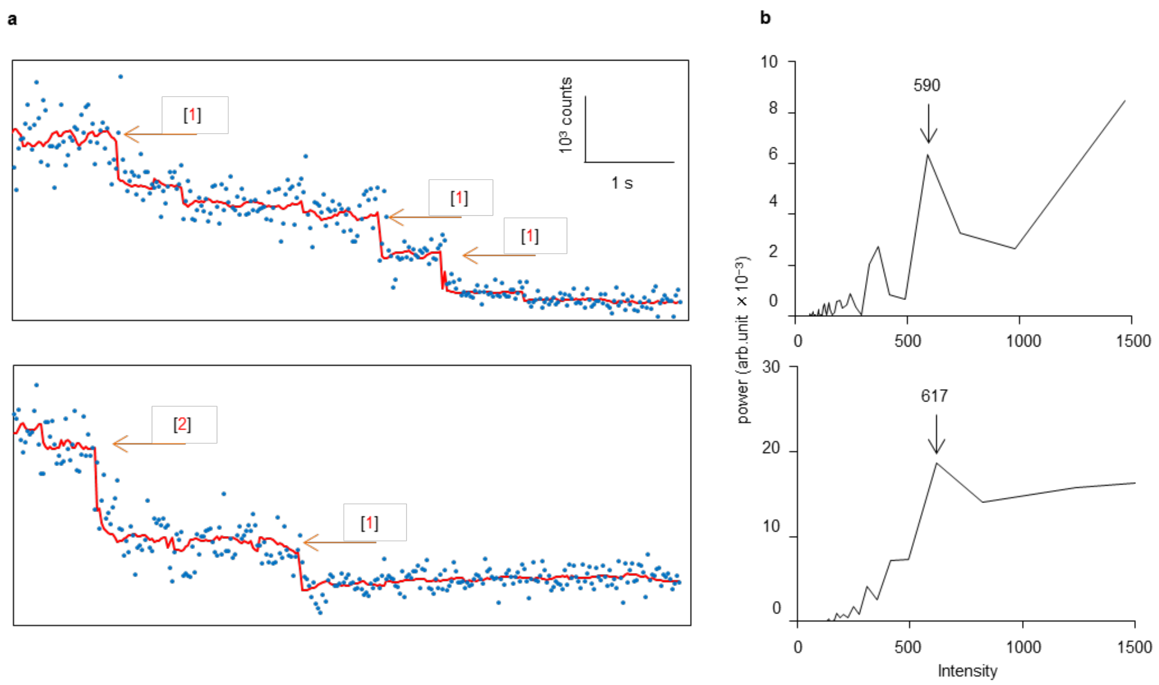

Figure S4 | Estimation of the number of AcrB-GFP molecules in single foci. The fluorescent intensities of AcrB-GFP foci in the presence of TolC were measured. Images were recorded with the exposure time of 33 ms for 10 s using TIRF microscopy. Fluorescent intensity per frame of a 10 × 10 pixels (500 × 500 nm) ROI centred at each fluorescent focus was monitored over time. The edge-detecting method of non-linear filtering (window = 15) was used to identify steps in the time-course of AcrB-GFP intensity. Calculating the distribution of the pairwise differences (Pairwise Difference Distribution Function, PDDF) and the power spectrum were performed using custom software written by LabVIEW2011 (National Instruments). (**a, b**) Stepwise photobleaching of single AcrB-GFP foci. Blue dots, the intensity of AcrB-GFP per frame; red line, output of the edge-detecting filtered intensity; orange arrows, the positions of predicted steps with measured a step size respectively; and red numbers, the number of bleached GFP molecules (**a**). Power spectra of the PDDF with arrows indicating step sizes of each photobleaching trace (**b**).

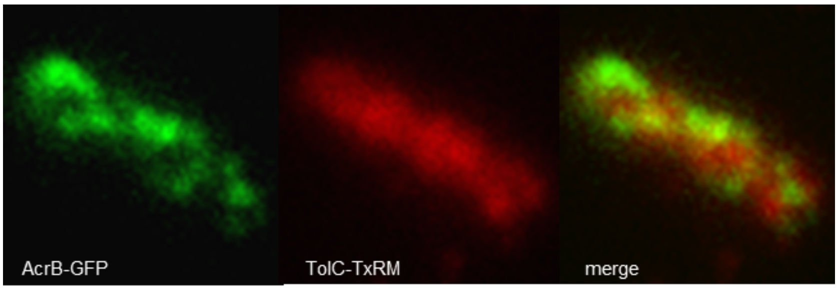

Figure S5 | Observation of TxRM-treated cells expressing AcrB-GFP and wild-type TolC. YKN17 cells (*acrB-gfp* Δ*tolC*) transformed with plasmid pKRB2100 (wild-type TolC) were grown in the presence of arabinose and treated with 1 μM TxRM. Images of the same fields taken with emission filters for GFP (left) or TxRM (middle) were merged (right).

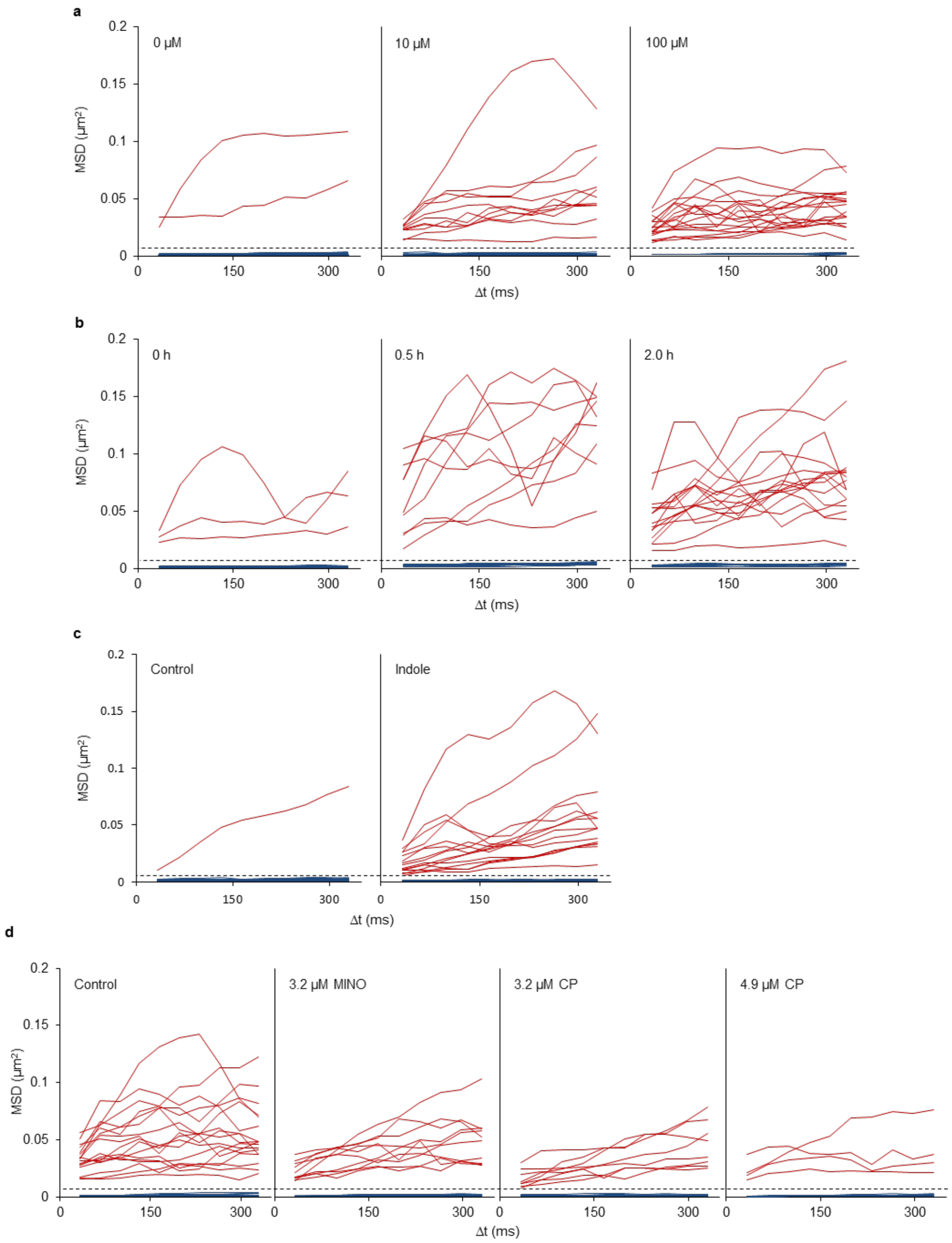

Figure S6 | Effect of the expression of AcrD on AcrB-GFP dynamics. MSD-Δ*t* plots of AcrB-GFP foci under various conditions are shown. Dotted line indicates the boundary MSD value at time 330 ms to define mobile (red lines) and stationary foci (blue lines). (**a**) Effect of the inducer concentration. The plasmid-borne *acrD* gene was induced with 0−100 μM arabinose for 2 h (n = 25). (**b**) Effect of induction time. The plasmid-borne *acrD* gene was induced with 100 μM arabinose for 0−2.0 h. (n = 25). (**c**) Effect of induction of the chromosomal *acrD* gene. The native *acrD* gene was induced with 4 mM indole for 2 h (n = 25). Cells were also treated with 1% dimethyl sulfoxide as a negative control. (**d**) Effects of the AcrB-specific substrates CP and MINO on MSDs of AcrB-GFP under AcrD-inducing conditions. The plasmid-borne *acrD* gene was induced with 100 μM arabinose for 2 h. Abbreviations: CP, chloramphenicol; MINO, minocycline (n = 25).

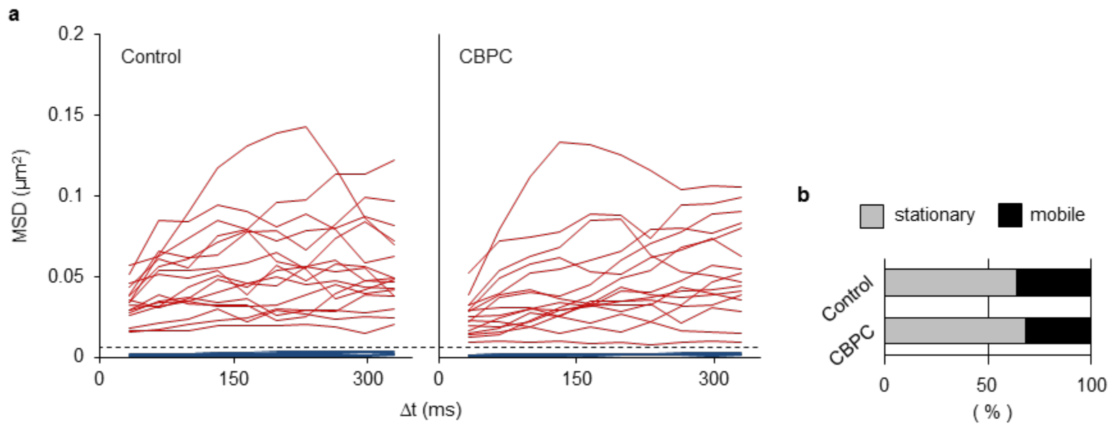

Figure S7 | Effect of an AcrD-specific substrate on transporter exchange. Carbenicillin (CBPC) was added to the culture for 1.5 h after *acrD* was induced with 100 μM arabinose. Cells were further incubated for 0.5 h. (**a**) MSD-Δ*t* plots of AcrB-GFP with (left) or without (right) 6.25 μM CBPC. (**b**) The fractions of stationary and mobile AcrB-GFP foci (n = 25) with or without CBPC.

Table S1 | Bacterial strains and plasmids used in this study.

	Relevant characteristics	Parent	Source or reference
***E. coli* strains**			
BW25113	wild type	W1485	Datsenko & Wanner, 2000
JW0451	Δ*acrB*::*kan*	BW25113	Baba *et al*., 2006
JW5503	Δ*tolC*::*kan*	BW25113	Baba *et al*., 2006
YKN12	*acrB-gfp*	BW25113	This study
YKN17	*acrB-gfp* Δ*tolC*::*kan*	BW25113	This study
MBRT02	*acrD-gfp*	BW25113	This study
**Plasmids**			
pTrcHisB	vector	–	Invitrogen
pDS1050	GFP fusion vector plasmid (C-terminal)	pTrcHisB	Hiremath *et al.*, 2015
pKRB2000	AcrB-GFP	pTrcHisB	This study
pKRB2010	AcrD-GFP	pTrcHisB	This study
pBAD24	vector	–	Guzman *et al.*, 1995
pKRB2050	AcrD	pBAD24	This study
pBAD33	vector	–	Guzman *et al.*, 1995
pKRB2053	AcrD	pBAD33	This study
pKRB2100	TolC	pBAD33	This study
pKRB2104	TolC-A269C	pBAD33	This study
pKD46	Red recombinase	–	Datsenko & Wanner, 2000
pCA24N	vector	–	Kitagawa *et al.*, 2005
pBaeR	His6-BaeR	pCA24N	Kitagawa *et al.*, 2005

Supplementary Videos Legends

Video 1 | Observation of single AcrB-GFP molecules expressed from the chromosomal gene of *tolC*$^+$ cells. This video shows the dynamics of TIRF-illuminated fluorescent foci formed by AcrB-GFP expressed from the chromosomal gene of strain YKN12 (*acrB-gfp*). Data were acquired at 30 frames per second. Scale bar, 1 μm.

Video 2 | Observation of single AcrB-GFP molecules expressed from the chromosomal gene of Δ*tolC* cells. This video shows the dynamics of TIRF-illuminated fluorescent foci formed by AcrB-GFP expressed from the chromosomal gene of strain YKN17 (*acrB-gfp* Δ*tolC*). Data were acquired at 30 frames per second. Scale bar, 1 μm.

Video 3 | Observation of single AcrB-GFP molecules expressed from the chromosomal gene of Δ*tolC* cells complemented with a plasmid encoding TolC. This video shows the dynamics of TIRF-illuminated fluorescent foci formed by AcrB-GFP expressed from the chromosomal gene of strain YKN17 (*acrB-gfp* Δ*tolC*) transformed with plasmid pKRB2100 carrying the *tolC* coding region downstream of the *araBAD* promoter, which was induced with 100 μM arabinose. Data were acquired at 30 frames per second. Scale bar, 1 μm.

Video 4 | Effect of an AcrB-specific substrate on the dynamics of single AcrD-GFP

molecules expressed from the chromosomal gene. This video shows the dynamics of TIRF-illuminated fluorescent foci formed by AcrD-GFP expressed from the chromosomal gene of strain MBRT02 (*acrD-gfp*), the native *acrD* promoter of which was induced with 4 mM indole for 2 h with (right) or without (left) an AcrB-specific substrate (4.9 μM chloramphenicol). Data were acquired at 30 frames per second. Scale bar, 1 μm.
